# Risk Factors for Radiation-Induced Keratoconjunctivitis Sicca in Dogs Treated with Hypofractionated Intensity-Modulated Radiation Therapy for Intranasal Tumors

**DOI:** 10.3390/ani15152258

**Published:** 2025-08-01

**Authors:** Akihiro Ohnishi, Soichirou Takeda, Yoshiki Okada, Manami Tokoro, Saki Kageyama, Shinya Mizutani, Yoshiki Itoh, Taketoshi Asanuma

**Affiliations:** 1 Department of Veterinary Medicine, Faculty of Veterinary Medicine, Okayama University of Science, Imabari 794-0085, Japan; a-oonishi@ous.ac.jp (A.O.); v19m099ts@ous.jp (S.T.); v19m040oy@ous.jp (Y.O.); v19m106tm@ous.jp (M.T.); v25mde1zc@ous.jp (S.K.); s-mizutani@ous.ac.jp (S.M.); 2 Chushikoku Animal Eye Clinic, Fukuyama 729-0111, Japan; yo-itoh@chushikoku-aec.com

**Keywords:** keratoconjunctivitis sicca, canine intranasal tumor, intensity-modulated radiation therapy, dosimetric analysis, radiation-induced ocular toxicity, dose threshold

## Abstract

Radiation-induced keratoconjunctivitis sicca (KCS) is a significant late complication in dogs receiving radiation therapy for intranasal tumors, particularly with hypofractionated intensity-modulated radiation therapy (IMRT). This retrospective case-control study was performed to identify anatomical and dosimetric risk factors for KCS in 15 canine patients treated with IMRT delivered in 4–6 weekly fractions of 8 Gy. Orbital structures were retrospectively contoured, and dose–volume metrics (D50) were calculated. Receiver operating characteristic (ROC) curve analysis and odds ratios were used to evaluate the associations between radiation dose and KCS development. Six dogs (33%) developed KCS within three months post-treatment. Statistically significant dose differences were observed between affected and unaffected eyes for the eyeball, cornea, and retina. ROC analyses identified dose thresholds predictive of KCS: 13.8 Gy (eyeball), 14.9 Gy (cornea), and 17.0 Gy (retina), with the retina showing the highest odds ratio (28.33). To ensure clinical relevance, KCS was diagnosed based on decreased tear production combined with corneal damage to ensure clinical relevance. This study proposes dose thresholds for ocular structures that may guide treatment planning and reduce the risk of KCS in canine patients undergoing IMRT. Further prospective studies are warranted to validate these thresholds and explore mitigation strategies for high-risk cases.

## Simple Summary

Radiation therapy is commonly used to treat nasal tumors in dogs, but it may cause eye-related complications, such as keratoconjunctivitis sicca (KCS), or “dry eye.” This condition can affect the quality of life of dogs due to eye discomfort and visual impairment. In this study, we analyzed the relationship between radiation doses to specific eye structures and the development of KCS in 15 dogs treated with high-dose, hypofractionated intensity-modulated radiation therapy (IMRT). We found that higher radiation doses to the eyeball, cornea, and retina were significantly associated with the occurrence of KCS. We also identified specific dose thresholds that can help predict the risk of KCS. These findings may help veterinarians design treatment plans that reduce the risk of ocular complications while still effectively treating nasal tumors in dogs.

## 1. Introduction

Radiation therapy (RT) is widely employed to treat canine and feline patients [1]. It is considered the gold standard for managing intranasal tumors and is frequently employed for this purpose [2]. While adenocarcinoma is the most commonly diagnosed nasal tumor, other histological types, including squamous cell carcinoma, undifferentiated carcinoma, transitional cell carcinoma, and sarcomas, have also been reported [3].

Veterinary RT requires general anesthesia to ensure proper immobilization of animals during treatment. Hypofractionated radiotherapy offers several advantages, including a reduced burden from repeated general anesthesia and decreased frequency of hospital visits [4,5]. These benefits make hypofractionated radiotherapy a promising treatment option.

Keratoconjunctivitis sicca (KCS), also known as dry eye syndrome, is a common ocular condition. KCS causes symptoms such as eye dryness, foreign body sensation, decreased vision, and an increased risk of infection. It is a chronic ophthalmologic condition characterized by decreased tear fluid volume, which can lead to corneal inflammation and corneal ulceration in severe cases [6]. KCS is also recognized to be associated with RT and frequently occurs as a radiation-induced injury during the treatment of canine intranasal tumors [7,8]. It is not merely a localized side effect, as it requires long-term administration of eye drops and can reduce visual function, negatively impacting quality of life.

Hypofractionated regimens are expected to result in a higher incidence of severe late complications than multifractionated regimens [9]. Although tolerable radiation doses have been reported in humans in assessments of risk of radiation injury [10], data for dogs remain limited. Doses may vary widely depending on the irradiation protocol, and no reports currently describe hypofractionated radiotherapy using high-dose intensity-modulated radiation therapy (IMRT).

In this study, we calculated the irradiation dose received by each ophthalmic structure based on computed tomography (CT) findings for intranasal tumors. Specifically, we: (1) grouped the cases according to CT findings and compared the doses received between groups; (2) compared the doses received by each ophthalmic structure in eyes that developed KCS and eyes that did not exhibit ophthalmologic symptoms and determined threshold values; and (3) examined the odds ratio (OR) for predicting KCS development based on CT findings and the identified threshold values for each structure.

## 2. Materials and Methods

### 2.1. Case Selection

This retrospective, case-controlled study reviewed the medical records of dogs with intranasal tumors undergoing RT with 4–6 weekly fractions of 8 Gy using IMRT between January 2020 and June 2024 at the Veterinary Education Hospital, Okayama University of Science. All dogs received standard clinical treatment, and retrospective analysis of clinical data was performed without the need for additional intervention. Inclusion criteria required continuous ophthalmologic examinations (Schirmer tear test [STT] and fluorescein staining test) performed before irradiation and up to 3 months post-irradiation. STT I (Eagle Vision, Memphis, TN, USA) was performed in our clinic by placing a standardized 5 × 35 mm strip of Whatman No. 41 filter paper in the ventral conjunctival fornix for 60 s without topical anesthesia. Fluorescein staining was performed by touching the lower eyelids with a sterile fluorescein strip. The patients were induced to blink several times to evenly distribute the dye over the ocular surface. KCS was suspected when STT values were below 15 mm/min and the fluorescein staining test was positive. However, diagnosis of KCS was not based solely on STT values. Since subclinical tear deficiency may not result in clinical signs or ocular surface damage, KCS was diagnosed only when decreased tear production was accompanied by fluorescein-positive corneal damage. This composite definition reflected clinically significant ocular toxicity. We assessed other ophthalmic signs, including hyperemia, mucoid discharge accumulation, and keratitis. Dogs with pre-existing ocular disease were excluded. Information retrieved from the medical records included breed, weight, sex, tumor histology, modified Adams stage, CT findings, development of KCS, and other ophthalmic complications.

### 2.2. Positioning and Planning CT

All dogs were positioned under general anesthesia. Anesthesia was induced with either propofol or alfaxalone, followed by endotracheal intubation, and maintained with sevoflurane inhalation during positioning and treatment. Dogs were routinely immobilized using a vacuum deformable mattress (Vac-LokTM Cushion, Toyo Medic Co., Ltd., Tokyo, Japan), a Uni-frame thermoplastic mask with an MT20100-type acrylic standard base plate (Toyo Medic Co., Ltd., Tokyo, Japan), and a bite block (Express Registration Materials, 3M Japan Limited, Tokyo, Japan) for both the planning CT and each RT session. All dogs were positioned in sternal recumbency, with forelimbs extending caudally and the elbows extended. Planning CT was performed using a 16-slice helical multidetector CT scanner (Aquilion Lightning, Canon Medical Systems, Otawara, Japan).

### 2.3. Original Radiation Planning

Treatment plans were generated using planning software (Monaco 6.1.4.0, Elekta Japan, Tokyo, Japan) employing a Monte Carlo equivalent algorithm. Pre- and post-contrast CT images were co-registered before contouring. Contouring was performed on the pre-contrast images with a slice thickness of 2 mm. The standard contouring technique involved gross tumor volume (GTV) delineation on contrast-enhanced CT in a bone window, with clinical target volume (CTV) expansion on a case-by-case basis, including frontal sinus fluid if present. Organs at risk (OARs) routinely contoured on the original plan included the eyeball, right eye (OD), left eye (OS), and brain. All dogs were prescribed 8 Gy in 4–6 fractions on a weekly schedule. The plan was normalized to ensure that 95% of the planning target volume (PTV) received 90% of the prescribed dose. While efforts were made to reduce exposure of the eyes, target coverage was prioritized. No specific efforts were made to limit the dose to orbital structures during the original planning.

### 2.4. Radiation Treatment

All dogs were anesthetized for treatment positioning and radiation delivery. Anesthesia was induced using either propofol or alfaxalone, followed by endotracheal intubation and maintained with sevoflurane in oxygen. Isocenter and beam arrangements were determined based on PTV locations. RT was delivered using 6 MV photons from a linear accelerator (Elekta Synergy, Canon Medical Systems, Otawara, Japan) employing a step-and-shoot technique. Before each RT session, sulfoquinovosylacylpropanediol (SQAP) (4 mg/kg) was injected intravenously as a radiosensitizing agent. RT was performed 15 min after injection of the radiosensitizing agent.

### 2.5. Follow-Up

The standard recommended follow-up for dogs with intranasal tumors involves monthly post-irradiation rechecks until adverse effects are observed. Advanced imaging was performed after an owner consented to participate. During follow-up visits, ophthalmologic examinations, including STT and fluorescein staining, were routinely conducted.

### 2.6. Contouring of Orbital Structures

For the dogs included in this study, duplicates of the orbital structure set were created using the Monaco planning system. Twelve additional structures were retrospectively created and contoured bilaterally for each dog: the cornea, retina, eyelid, lens, lacrimal gland, and third eyelid gland bilaterally. These structures were contoured in the axial plane on contrast-enhanced scans in a soft tissue window as follows:

1. Cornea: Contoured rostral to the anterior chamber. As the distinction between the retinal terminus and corneal origin was not clearly visible, a subjective choice was made based on the presumed location of the iridocorneal angle (with reference to the medial and lateral aspects of the lens) [11].
2. Retina: The “retina-choroid-sclera complex” appears as a single hyperdense curved line bracketed by the rectus muscle and is delineated from the posterior face of the vitreous chamber in pre-contrast studies [12].
3. Eyelid: The skin on the cranial side of the eye was contoured.
4. Lens: contoured as a biconvex hyperdense structure distinguishable on pre- and post-contrast CT images [12].
5. Lacrimal gland and third eyelid gland: contoured as visualized on contrast-enhanced CT images [9].

**Figure 1:**
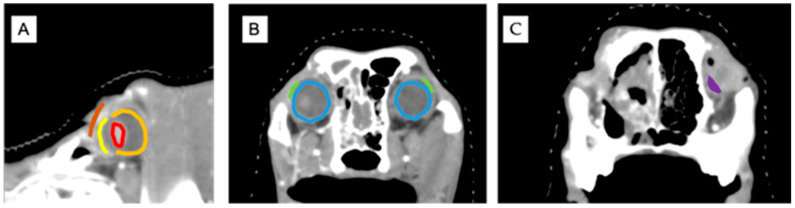
Representative CT images showing contoured ocular structures used for dose–volume histogram analysis. (**A**) Sagittal plane demonstrating the cornea (yellow), lens (red), retina (orange), and eyelid (brown). (**B**) Transversal plane centered at the level of the eyeballs, highlighting the eyeballs (blue) and lacrimal glands (green). (**C**) Transversal plane cranial to the eyeballs, showing the contoured third eyelid (purple).

Figure 1 illustrates the anatomical contours used in this study, including the eyeball, cornea, retina, third eyelid, and optic nerve region. These structures were delineated on both axial and sagittal CT images for accurate dosimetric analysis.

### 2.7. Dose Calculation

The orbital structures were utilized with the original plan parameters to generate the dose distribution for the new structure set. For each dog, dose distribution was verified to be identical for all the original structures. Using dose–volume histogram for each orbital structure, the dosimetric parameters evaluated were the radiation doses at which 50% of the structures received the specified dose (D_50_).

### 2.8. Statistical Analyses

Descriptive statistics were used for continuous data, assuming normality of mean values. The two groups were analyzed for differences in D_50_ values of the orbital structures. Variables were first analyzed for normality using the Shapiro-Wilk test. The Mann–Whitney U test was used for parametric and nonparametric data. The threshold value for D_50_ of each structure associated with KCS development was determined using receiver operating characteristic (ROC) curve analysis. This threshold was subsequently set as the cutoff value, and the odds ratio (OR) was calculated. To evaluate the significance of each OR, a chi-square test was conducted. Statistical significance was set at *p* < 0.05. All statistical analyses were performed using GraphPad Prism version 9.5.0 (GraphPad Software Inc., San Diego, CA, USA).

## 3. Results

Fifteen dogs were treated during the study period. All patients completed the radiation protocol within 5–6 weeks. During the follow-up period, eyes exhibiting conjunctival hyperemia were diagnosed with conjunctivitis (VRTOG Grade 1) [13], whereas eyes with STT values of ≤10 mm/min and positive fluorescein staining were diagnosed with KCS. Acute ocular toxicity observed in the present study was limited to grade 2, with no grade 3 or higher toxicities observed. Six dogs (33%) developed KCS, one bilaterally and the remainder unilaterally. Three dogs (20%) developed conjunctivitis, one bilaterally and the others unilaterally. Eight dogs met the inclusion criteria for the control group, resulting in a total of six KCS eyes and 20 control eyes, excluding four eyes with conjunctivitis.

Dogs treated with radiotherapy were grouped according to CT findings of intranasal tumors based on the modified Adams classification, presence of orbital invasion, and subcutaneous invasion of the frontal sinus. All cases were summarized according to the CT findings and ophthalmologic complications, considering whether the affected eye was ipsilateral or contralateral to the tumor (Table 1).

**Figure 2:**
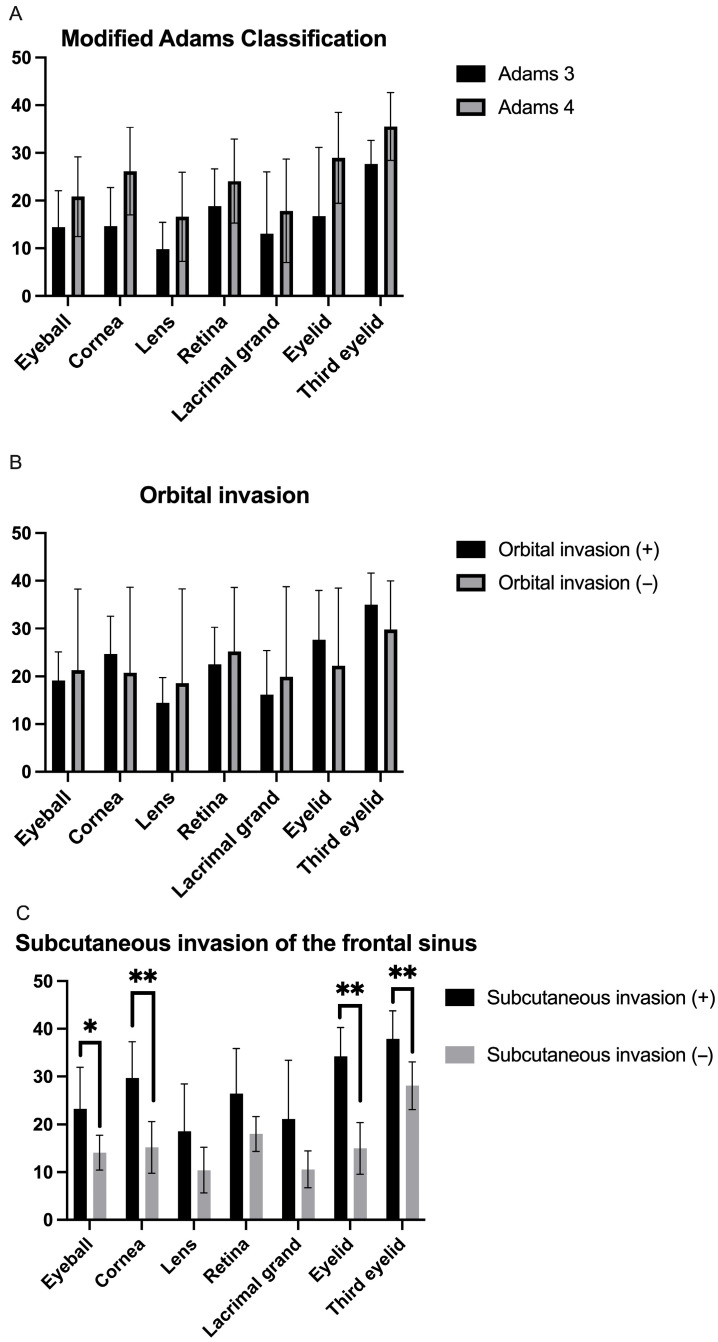
Comparison of radiation doses to ocular and periocular tissues according to (**A**) modified Adams classification, (**B**) presence or absence of orbital invasion, and (**C**) subcutaneous invasion of the frontal sinus. Data are expressed as means ± standard deviations. * *p* < 0.05, ** *p* < 0.01.

The doses administered to the ocular and periocular tissues in each group are summarized in Figure 1. No significant difference in dose was found for ocular and periocular tissues in the modified Adams classification or in the group distinguished by the presence or absence of orbital invasion (Figure 2A,B). However, significant differences were observed in the eyeball, cornea, eyelid, and third eyelid gland in the group distinguished by the presence or absence of subcutaneous invasion of the frontal sinus (Figure 2C).

**Figure 3:**
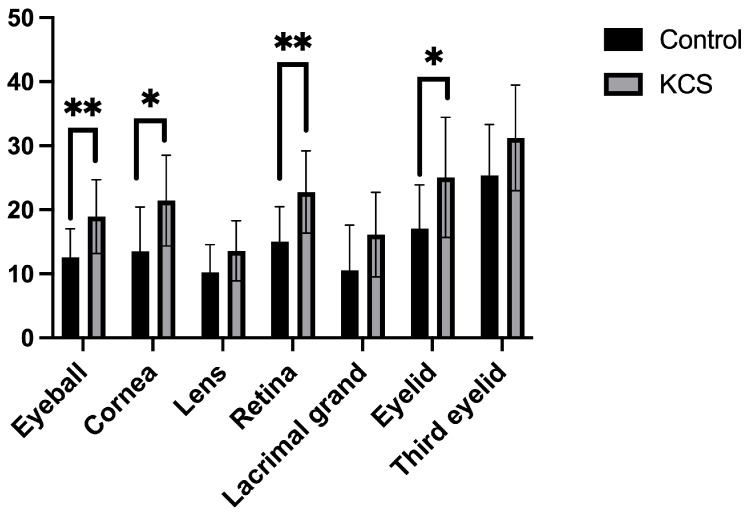
Comparison of radiation doses to ocular and periocular tissues between eyes that developed keratoconjunctivitis sicca (KCS) and those without adverse effects (control group). Data are expressed as means ± standard deviations. * *p* < 0.05, ** *p* < 0.01.

Radiation doses to ocular and periocular tissues were compared between eyes that developed KCS and those without adverse effects (Figure 3). In these comparisons, eyes that developed conjunctivitis were excluded, and eyes without ophthalmologic complications were considered the control group, whereas those diagnosed with KCS were considered the KCS group. When comparing control and KCS groups, significant differences were observed in the D_50_ values for the eyeball, cornea, retina, and eyelid.

**Figure 4:**
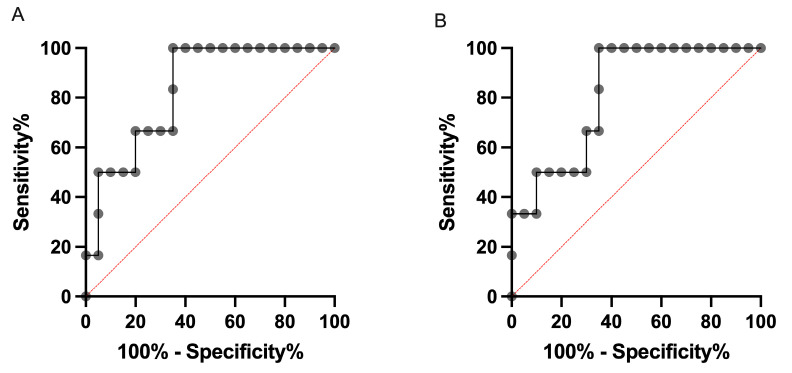

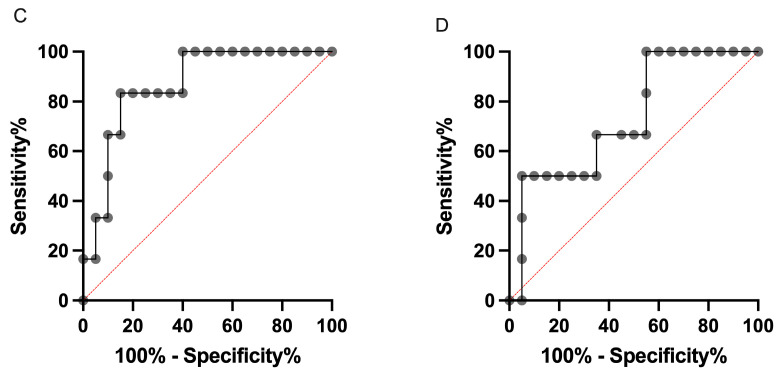
Receiver operating characteristic (ROC) curves for D_50_ as a predictor of keratoconjunctivitis sicca (KCS) in different ocular structures: (**A**) eyeball, (**B**) cornea, (**C**) retina, and (**D**) eyelid.

As shown in Figure 4, ROC analysis of D_50_ for the eyeball (AUC = 0.83, 95% confidence interval [CI]; 0.67–1.00, *p* = 0.015), cornea (AUC = 0.82, 95% CI; 0.64–0.99, *p* = 0.020), and retina (AUC = 0.87, 95% CI; 0.72–1.00, *p* = 0.007) showed significant predictive power for the development of KCS, whereas that for the eyelid did not reach statistical significance (AUC = 0.73, 95% CI; 0.51–0.96, *p* = 0.088).

For eyeballs, corneas, and retinas with significant differences in ROC analyses, cutoff values were determined using the Youden index, and ORs were calculated using these cutoffs as threshold values. ORs were calculated for subcutaneous invasion of the frontal sinus. Subcutaneous invasion of the frontal sinus had an OR of 2.00 (95% CI: 0.21–31.7), and the chi-square test showed no statistically superior association (χ^2^ = 0.2800, *p* = 0.5967). The OR was calculated using D_50_ = 13.8 Gy as the dose threshold to the eyeball. The OR was 9.286 (95% CI: 1.195–116.1), and the chi-square test showed a statistically superior association (χ^2^ = 4.338, *p* = 0.0373). The OR for cornea using D_50_ = 14.9 Gy as the dose threshold was 9.286 (95% CI: 1.195–116.1), and the chi-square test showed a statistically superior association (χ^2^ = 4.338, *p* = 0.0373). The OR for the retina using D_50_ = 17.0 Gy as the dose threshold was 28.33 (95% CI: 2.872–345.3), and the chi-square test showed a statistically superior association (χ^2^ = 10.12, *p* = 0.0015). The OR for the eyelid using D50 = 15.9 Gy as the dose threshold was 4.091 (95% CI: 0.5512–52.8), and the chi-square test showed no statistically significant association (χ^2^ = 1.565, *p* = 0.2109).

## 4. Discussion

We successfully identified pathoanatomical risk factors associated with the development of KCS in dogs receiving hypofractionated IMRT for intranasal tumors and established irradiation dose thresholds for orbital structures.

Radiation-induced injury is a critical concern in RT, and ophthalmologic disorders caused by radiotherapy of intranasal tumors have been extensively studied because of their impact on quality of life. These disorders can result from dysfunction of the lacrimal gland, third eyelid, corneal surface, and eyelids, leading to an inadequate tear film [2]. Among these, KCS is a radiation-induced injury that exerts a particularly significant negative impact on a patient’s quality of life due to the elevated potential for severe and painful corneal ulcers resulting from corneal inflammation [14,15].

Clinical–dosimetric relationships between clinical symptoms and radiation dose for KCS have been reported in humans and dogs and are considered valuable for predicting radiation-induced injury [8]. However, differences in treatment protocols must be considered when evaluating the development of radiation injuries. Multifractionated and hyperfractionated irradiation protocols show comparable treatment outcomes. Although hypofractionated irradiation may carry a higher risk of radiation-induced injury, few studies have examined its specific effects [16].

KCS is defined as inflammation of the cornea and conjunctiva accompanied by quantitative and qualitative abnormalities of the lacrimal fluid. However, previous studies’ diagnoses of radiation-induced KCS have mainly relied on STT [8]. As this alone is insufficient for a definitive diagnosis of KCS, the present study sought to detect KCS as keratoconjunctivitis with decreased tear fluid. Specifically, we considered decreased tear production in conjunction with corneal surface injury, detected by fluorescein-positive staining, as essential for the diagnosis of clinically relevant KCS. This approach avoids overestimation due to transient tear suppression and ensures that only cases with potential impact on vision and quality of life are included. Some reports indicate that the onset of KCS in dogs as a radiation-induced injury from hypofractionated irradiation occurs in the acute phase, whereas others report that it occurs 3–4 months later [4]. In this study, we examined the onset of KCS in the acute phase (within three months) because the cornea, conjunctiva, and eyelids can be damaged acutely by radiation, although some reports indicate that the lacrimal gland and third eyelid gland damage can occur after three months [17].

In the present study, exposure to sites other than the lacrimal and third eyelid glands was significantly associated with KCS development. This finding is particularly important because the retina is not directly involved in tear production or corneal protection. One possible explanation for this association is that the retinal dose serves as an indicator for the overall ocular radiation exposure. Alternatively, retinal damage may indirectly contribute to the development of KCS through neurogenic mechanisms or alterations in ocular blood flow.

Subcutaneous invasion of the frontal sinus has been identified as a potential pathianatomical risk factor for KCS development. Although the OR for this factor was not statistically significant, further investigation in a larger study is warranted. Subcutaneous invasion may necessitate larger treatment volumes, potentially increasing the radiation dose to nearby ocular structures.

The dose thresholds established in this study for the eyeball, cornea, and retina provide valuable guidance for radiation oncologists when planning hypofractionated IMRT for canine intranasal tumors. By adhering to these thresholds, clinicians may reduce the incidence of KCS while maintaining effective tumor control. It is important to note that these thresholds should be considered in conjunction with other clinical factors and not as absolute limits.

Our study has several limitations. First, the sample size is relatively small (*n* = 15), which may affect the generalizability of the findings. Further studies involving larger populations are warranted to validate the identified dosimetric thresholds. Second, the follow-up period was limited to three months, which may not have captured late-onset KCS cases. Future studies with longer follow-up periods are required to assess the development of both acute and chronic KCS. Third, although we were unable to define a significant dose as a threshold in this study, the results suggest that eyelid exposure may play a significant role in KCS development, possibly due to the presence of the meibomian glands in the eyelids. Radiation exposure to these glands may affect the development of KCS by causing qualitative abnormalities in the lacrimal fluid due to dysfunction of the meibomian glands. Evaluation of the meibomian gland as a follow-up to radiotherapy may thus be necessary to predict the risk of KCS in the future. Finally, while the radiosensitizer SQAP was used, the extent to which its effect contributed to the incidence of KCS remains unclear. Human clinical data provide limited evidence that radiosensitizers increase the risk of radiation-induced KCS. In this study, the impact of SQAP use on the incidence of KCS remains unclear. Larger studies and analyses with control groups are needed. Currently, no evidence indicates that the sensitizers exacerbate KCS. However, as radiosensitizers like SQAP enhance tumor oxygenation and modulate the local microenvironment, it is essential to evaluate their influence on surrounding normal tissues, particularly in organs at risk such as the eye. The potential for increased sensitivity to radiation-induced damage in ocular structures warrants further investigation in prospective studies.

Additionally, the contouring of the orbital structures was performed retrospectively, which may have introduced some variability. However, we attempted to minimize this by using standardized contouring protocols, with a single experienced doctor performing all contouring.

Despite these limitations, our study provides valuable insights into the dosimetric and pathoanatomical risk factors for KCS development in canines undergoing hypofractionated IMRT for intranasal tumors. The identified dose thresholds and anatomical considerations can inform future prospective studies and guide clinical decision-making.

In veterinary medicine, radiation therapy is often administered to patients with limited expected survival times due to the advanced stage of disease at diagnosis. As a result, long-term follow-up to assess late-onset radiation-induced complications is frequently not feasible. However, future studies incorporating follow-up periods exceeding three months are essential to better understand the mechanisms and progression of radiation-induced damage, including the development of KCS and other late ocular effects.

## 5. Conclusions

We identified significant dosimetric and anatomical risk factors associated with the development of KCS in canines undergoing hypofractionated IMRT for intranasal tumors. We have established dose thresholds for the eyeball, cornea, and retina that are predictive of KCS development, with the retina emerging as a critical structure, showing the highest OR for KCS prediction.

These findings have important implications for radiation treatment planning and may help reduce the incidence of KCS in canine patients. By adhering to the identified dose thresholds and considering anatomic factors such as subcutaneous invasion of the frontal sinus, veterinary radiation oncologists can potentially mitigate ocular complications while preserving effective tumor control.

Future prospective studies with larger sample sizes and extended follow-up periods are warranted to validate these findings and to further refine our understanding of radiation-induced KCS in canines. Additionally, investigating potential preventive measures for high-risk patients and exploring adaptive planning techniques to minimize ocular dose may further improve outcomes for canines undergoing RT for intranasal tumors.

## Figures and Tables

**Table 1:** Summary of patient characteristics and treatment parameters. “Ipsilateral” and “Contralateral” refer to the side of the eye relative to the primary tumor. “+” indicates presence, and “None” indicates absence of the finding or complication. Modified Adams classification was used for tumor staging. KCS was diagnosed based on a Schirmer tear test < 15 mm/min and positive fluorescein staining.

Patient	Histological Diagnosis	Modified Adams Classification	Orbital Invasion	Subcutaneous Invasion of the Frontal Sinus	Opthalmologic Complication—Ipsilateral Eye	Opthalmologic Complication—Contralateral Eye
1	Nasal adenocarcinoma	3	+	None	None	None
2	Undifferentiated carcinoma	4	None	+	conjunctivitis	conjunctivitis
3	Nasal sarcoma	4	+	+	conjunctivitis	None
4	Nasal adenocarcinoma	4	+	+	None	None
5	Squamous cell carcinoma	4	+	+	None	None
6	Nasal adenocarcinoma	4	+	+	conjunctivitis	KCS
7	Undifferentiated carcinoma	4	+	+	KCS	None
8	Nasal carcinoma	4	+	+	KCS	None
9	Chondrosarcoma	3	None	None	None	None
10	Nasal adenocarcinoma	4	+	None	None	None
11	Nasal adenocarcinoma	4	+	+	None	None
12	Nasal carcinoma	4	+	None	KCS	KCS
13	Nasal carcinoma	3	+	+	KCS	None
14	Nasal carcinoma	4	None	None	None	None
15	Transitional carcinoma	4	+	None	None	None

## Data Availability

The data are not publicly available due to privacy and ethical restrictions. Anonymized datasets may be available from the corresponding author upon reasonable request and with approval from the institutional ethics board.

## Abbreviations

The following abbreviations are used in this manuscript:

KCS	Keratoconjunctivitis sicca
IMRT	Intensity-modulated radiation therapy
STT	Schirmer tear test
CT	Computed tomography
RT	Radiation therapy
ROC	Receiver operating characteristic
SQAP	Sulfoquinovosylacylpropanediol
OR	Odds ratio
CI	Confidence interval
