# Correction: Ohnishi et al. Risk Factors for Radiation-Induced Keratoconjunctivitis Sicca in Dogs Treated with Hypofractionated Intensity-Modulated Radiation Therapy for Intranasal Tumors. *Animals* 2025, *15*, 2258

**DOI:** 10.3390/ani15243646

**Published:** 2025-12-18

**Authors:** Akihiro Ohnishi, Soichirou Takeda, Yoshiki Okada, Manami Tokoro, Saki Kageyama, Shinya Mizutani, Yoshiki Itoh, Taketoshi Asanuma

**Affiliations:** 1 Department of Veterinary Medicine, Faculty of Veterinary Medicine, Okayama University of Science, Imabari 794-0085, Japans-mizutani@ous.ac.jp (S.M.); 2 Chushikoku Animal Eye Clinic, Fukuyama 729-0111, Japan; yo-itoh@chushikoku-aec.com

**Addition of an Author**

Dr. Shinya Mizutani was not included as an author in the original publication [1]. The corrected Author Contributions statement appears here. The authors state that the scientific conclusions are unaffected. This correction was approved by the Academic Editor. The original publication has also been updated.

**Author Contributions:** Conceptualization, A.O. and T.A.; methodology, A.O., S.T., Y.O., M.T. and S.K.; formal analysis, A.O.; investigation, A.O.; resources, Y.I.; writing—original draft preparation, A.O.; writing—review and editing, all authors; visualization, S.M.; supervision, T.A. All authors have read and agreed to the published version of the manuscript.
